# Radiosurgery of limited brain metastases from primary solid tumor: results of the randomized phase III trial (NCT02355613) comparing treatments executed with a specialized or a C-arm linac-based platform

**DOI:** 10.1186/s13014-023-02216-5

**Published:** 2023-02-07

**Authors:** Marta Scorsetti, Pierina Navarria, Luca Cozzi, Elena Clerici, Luisa Bellu, Davide Franceschini, Antonio Marco Marzo, Ciro Franzese, Valter Torri, Giacomo Reggiori, Francesca Lobefalo, Luca Raspagliesi, Luca Attuati, Federico Pessina, Andrea Franzini, Piero Picozzi, Stefano Tomatis

**Affiliations:** 1 Radiotherapy and Radiosurgery Department, IRCCS Humanitas Research Hospital, Via Manzoni 56, 20089 Rozzano, Milan Italy; 2 Department of Biomedical Sciences, Humanitas University, Pieve Emanuele, Milan Italy; 3 Oncology Department, IRCCS Istituto Mario Negri, Milan, Italy; 4 Neurosurgery Department, IRCCS Humanitas Research Hospital, Rozzano, Milan Italy

**Keywords:** Randomized phase 3 trial, Radiosurgery, Gamma Knife-radiosurgery, Linac-based radiosurgery, Radionecrosis, Brain metastases

## Abstract

**Background:**

Comparative prospective data regarding different radiosurgery (SRS) modalities for treating brain metastases (BMs) from solid tumors are not available. To investigate with a single institute phase III randomized trial whether SRS executed with linac (Arm-B) is superior to a dedicated multi-source gamma-ray stereotactic platform (Arm-A).

**Methods:**

Adults patients with 1–4 BMs from solid tumors up to 30 mm in maximum diameter were randomly assigned to arms A and B. The primary endpoint was cumulative incidence of symptomatic (grade 2–3) radionecrosis (CIRN). Secondary endpoints were local progression cumulative incidence (CILP), distant brain failure, disease-free survival (DFS), and overall survival (OS).

**Results:**

A total of 251 patients were randomly assigned to Arm-A (121) or Arm-B (130). The 1-year RN cumulative incidence was 6.7% in whole cohort, 3.8% (95% CI 1.9–7.4%) in Arm-B, and 9.3% (95% CI 6.2–13.8%) in the Arm-A (*p* = 0.43). CIRN was influenced by target volume irradiated only for the Arm-A (*p* << 0.001; HR 1.36 [95% CI 1.25–1.48]). Symptomatic RN occurred in 56 cases at a median time of 10.3 months (range 1.15–54.8 months), 27 in the Arm-B at a median time of 15.9 months (range 4.9–54.8 months), and 29 in the Arm-A at a median time of 6.9 months (1.2–32.3 months), without statistically significant differences between the two arms. No statistically significant differences were recorded between the two arms in CILP, BDF, DFS or OS. The mean beam-on time to deliver SRS was 49.0 ± 36.2 min in Arm-A, and 3.1 ± 1.6 min in Arm-B.

**Conclusions:**

Given the technical differences between the treatment platforms investigated in this single-institution study, linac-based SRS (Arm-B) did not lead to significantly lower grade 2–3 RN rates versus the multi-source gamma-ray system (Arm-A) in a population of patients with limited brain metastases of small volume. No significant difference in local control was observed between both arms. For Arm-B, the treatment delivery time was significantly lower than for Arm-A.

*Trial registration*: ClinicalTrials.gov Identifier NCT02355613.

**Supplementary Information:**

The online version contains supplementary material available at 10.1186/s13014-023-02216-5.

## Introduction

The occurrence of brain metastases (BMs) is a challenging issue affecting about 20–40% of cancer patients [1]. In the last years, the number of brain metastatic patients has been growing due to the more effective systemic therapies, resulting in a better control of extra-cranial disease, and the wide use of brain MRI as diagnostic imaging [2–10]. Formerly, whole-brain radiation therapy (WBRT) has been employed as the most frequent therapeutic modality. More recently, stereotactic radiosurgery (SRS) was introduced to manage the treatment of patients with limited BMs, based on the results of 4 large phase 3 trials that randomized patients to receive SRS alone or WBRT plus SRS [11–15]. The significant increase in neurocognitive impairment using WBRT, without survival benefit, has made SRS the primary treatment choice for patients with limited BMs. SRS was initially developed in 1951 using a multi-source gamma-ray delivery platform although devices using cobalt sources came into clinical use around 1967–1968 [16]. A high dose conformity and a rapid fall-off dose close to the tumor characterize these treatments, and this platform remains a very commonly used modality for SRS. Generally, a frame system for patient immobilization is employed, although the most recent commercial version of the multi-source platform made the delivery of frameless SRS possible. Linear accelerator-based SRS expanded as an alternative in the 1980s [17]. It has become increasingly common, likely due to the easier use, the employ of a frameless system, the significant SRS delivery time reduction, and the lowered expense. Comparable brain control between the two modalities has been recorded [18, 19]. However, a higher risk of radionecrosis (RN) incidence was reported for the gamma-ray platform with respect to the linac-based procedures, above all when large BMs are treated [20–22]. The development of RN after stereotactic treatments is of concern. To date, prospective randomized trials comparing the two modalities are not yet available, but published data showed that the occurrence of RN is correlated with the volume of the irradiated normal brain; the risk of RN increases to 60% when at least 10 cm^3^ of normal brain tissue receives doses greater than 12 Gy [23–25]. Due to the lack of investigations on this issue, we designed a double-arm phase III trial comparing these two modalities for SRS. The primary aim was to assess the rate of symptomatic radionecrosis of the two SRS modes.

## Methods and materials

### Study design and patients

The present is a single institute prospective randomized, double-arms phase 3 trial approved by our institutional review board, registered at the ClinicalTrials.gov site with the number NCT02355613. The trial was approved by the Institutional Review Board, and it was monitored by the institutional Data and Safety Monitoring Board. The study aimed to compare SRS treatments executed on a Gamma-Knife Perfexion unit (Elekta AB, Stockholm, Sweden), Arm-A, or an Edge treatment system (Varian Medical Systems, Palo Alto, USA), Arm-B. All patients provided written informed consent to the treatment and the use of their data for scientific purposes. Eligible patients were aged 18–85 years, Karnofsky performance status (KPS) ≥ 70, histo-pathologically confirmed primary solid tumors, 1–4 BMs up to 30 mm in maximum diameter, recursive partitioning analysis (RPA) class 1–2, estimated survival ≥ 3 months as from disease specific-grade prognostic assessment (DS-GPA) score. Patients with a primary diagnosis of small cell lung cancer (SCLC), hematologic malignancy, pregnancy or prior whole brain radiotherapy were excluded from the enrolment. The administration of bevacizumab (possibly included in some chemotherapy schemes) for at least two months after radiosurgery was another exclusion criterion since potentially altering the RN patterns. Patients could have an active extra-cranial disease, and a systemic agents treatment has been permitted while in the study. The primary endpoint was the occurrence of symptomatic RN in our patients expressed as cumulative incidence (CIRN). Symptomatic RN was defined as grade 2 (moderate symptoms; corticosteroids indicated), grade 3 (severe symptoms; intervention indicated), and grade 4 (severe symptoms; immediate intervention indicated) as for Common Terminology Criteria for Adverse Events (CTCAE) version 3.0. Radiologically, RN was defined as the radiographic evidence of lesion on postcontrast T1MRI not visible on T2MRI sequences (T1/T2 mismatch), absence of perfusion on perfusion MRI, and/or absence of uptake on 11CMETPET. Secondary endpoints were cumulative incidence of local progression (CILP), distant brain failure (DBF), disease-free survival (DFS), and overall survival (OS). CILP was evaluated (per-lesion) measuring lesion volume variation according to the Response Assessment in Neuro-Oncology (RANO) Working Group [26]. DBF was defined as the absence of new brain lesions. DFS and OS were registered to measure the progression and death rate.

The total beam-on time was recorded as complementary endpoints as efficiency indicators. The total treatment time was not effectively recorded for all patients and will not be reported.

### Study design and randomization

The primary endpoint was the cumulative incidence of patients experiencing symptomatic RN (grade 2–4). A superiority design was assumed. Symptomatic RN was expected at around 10% at one year for Arm-A. Considering a rate of ~ 1% for Arm-B, 250 patients were required for the study to have a power of 80% to demonstrate this difference with a two-sided *p* < 0.05 by the χ^2^ test. The original trial design required performing the statistical analysis on the intent-to-treat principle. Patients were randomly assigned in a two-arm design (1:1) using a permuted block design employing a platform developed by the institutional clinical trial team. Patients were stratified by age (≤ 65 years vs > 65 years), presence of extra-cranial metastases (no *vs* yes), and the number of BMs (one *vs* two to four). The randomization was generated by the study data manager blindly to the clinical investigators.

### Treatment regimens

Concerning patient immobilization and treatment planning imaging, in Arm-A, a Leksell frame system was applied, and a volumetric MRI was performed. The target volume was defined as the contrast-enhancing tumor on the volumetric T1MRI. Prescription doses were 24 Gy for BMs ≤ 20 mm, and 20 Gy for lesions 21–30 mm, delivered at the 50% isodose line. No margins from target volume have been applied, and no image-guided performed before SRS treatment. The radiation dose was delivered with multi-isocenter plans so that the 50% isodose line conformed to 100% of the target volume. A basal CT scan without contrast and a post-contrast volumetric T1MRI were acquired for treatment planning in Arm-B. All the patients were immobilized with an open-face thermoplastic mask. The gross tumor volume (GTV) was defined as the contrast-enhancing tumor on the volumetric T1MRI. The planning target volumes (PTV) were generated by adding an isotropic margin of 2 mm from GTV. All plans were optimized with multiple isocentres (one per lesion) except for the cases where the separation between different metastases was inferior to 3 mm. In these cases, a shared isocentre was used. A single dose of 24 Gy was prescribed at the mean dose to PTV for metastases with a diameter ≤ 20 mm, while a dose of 20 Gy would have been prescribed for BMs with a diameter of 21–30 mm. The planning objective was that > 95% of PTV received 95% of the prescribed dose (PTV V95% > 95%) and that > 98% of GTV covered by 98% of the prescribed dose. SRS was delivered on an unit equipped with the high definition multileaf collimator with a leaf thickness at the isocentre in the target region of 2.5 mm. The Volumetric Modulated Arc Therapy technique was used, and flattening filter-free beams of 6 or 10 MV were selected for all patients. Image-Guided RT (IGRT) was performed with kV-CBCT, and patient repositioning was provided in six dimensions using an integrated six degrees of freedom treatment couch. The Optical Surface Monitoring Solution (OSMS, Varian Medical Systems, Palo Alto, US) performed real-time patient tracking during the delivery. For both arms, dose constraints to the organs at risk were the following: brain stem: V_12Gy_ < 10 cm^3^, optical nerves: D_2%_ < 8 Gy, chiasm: D_2%_ < 12 Gy (optimal: 8 Gy), cochlea: mean dose ≤ 4.5 Gy. Plan quality was further assessed through the RTOG Conformity Index $CI\;RTOG = PIV/TV$, the Paddick Conformity Index $PCI = TTV^{2} /(TV \cdot PIV$), the gradient index $GI = V_{50\% PI} /PIV$, the homogeneity index $HI = \left( {D_{max} - D_{min} } \right)/D_{mean}$ HI where PIV is the volume covered by the prescription isodose, TV is the volume of the target, TTV is the volume of the PTV covered by the prescription isodose, and V_50%PI_ is the volume covered by 50% of the prescription isodose. The near-to-maximum dose in the target was recorded as well. Dosimetric data were compared with 2-sided t-tests. Corticosteroids were administered, if needed, during the treatment and tapered in stable or improving patients as soon as possible. During the steroid administration, glucose blood levels were monitored once a week. Anticonvulsants were prescribed with the lower efficacy dose only in patients with a history of at least one seizure; the first choice drug was levetiracetam due to a lower incidence of toxicity and the lack of significant drug interactions (especially with chemotherapeutic agents). Other anticonvulsants such as topiramate, lamotrigine, or lacosamide were used when levetiracetam was not well tolerated. Due to the potential hepatotoxicity of anticonvulsant therapy, hepatic functionality was tested every two months.

### Follow-up and outcomes

Clinical and radiological evaluations have been performed at baseline, one month after treatment, and every 3 months after that, or until disease progression; they included history and physical examination, blood tests, and KPS. Hematologic and non-hematologic toxicities were graded according to Common Terminology Criteria for Adverse Events version 3.0. Brain MRI was performed aiming to differentiate post-treatment RN or disease progression. Methionine-CT/PET was performed in those cases with doubt images. An expert neuro-radiologist blindly assessed the response. Local recurrences were treated, and treatments could include surgery, SRS, or systemic agents if feasible. New distant BMs distinct from the treated sites were treated with SRS or WBRT in cases of leptomeningeal diffusion.

### Statistical analysis

Time to RN was defined as starting from the day of SRS up to the date of the MRI evaluation. Time to progression was defined as the interval from the date of SRS to the documentation of progression and evaluated for CNS progression (DBF), local progression and DFS, according to the different kinds of failures. Local progression and RN were computed on a per-lesion basis, whereas all other analyses were on a per-patient basis. OS was defined as the interval from SRS to death of any cause. The impact of RN and local progression was estimated by competing risk analysis and cumulative incidence approach considering death as the competing event [27, 28].

The difference in time to RN and local progression was compared between treatment arms using the method developed by Gray [29]. BDF, DFS, and OS were tested between the two study arms by the logrank test.

Variables considered were prescribed dose, number, and the dimension of CNS lesions. As further clinical predictors, gender, KPS score (1 for 70, 2 for 80, and 3 for 90–100), histology of primary tumor (non-lung vs lung metastases), stage of disease (1 for stages I and II, and 2 for stages III and IV), presence of extra-cranial metastases (yes/no), RPA class (I vs II), presence of systemic therapy (yes/no), number of BMs (1 for single lesions, 2 for more), and dose prescription were considered as categorical variables. Target volume and diameter were treated as continuous variables.

For each variable, HR and *p* values for BDF, DFS, and OS were computed by means of univariate Cox regression. The Fine and Gray method was applied to test predictors of CIRN and CILP.

For all endpoints, *p* ≤ 0.05 (two-sided) was defined as the significance threshold [30].

All analyses were conducted using the Stata software (StataCorp. 2017. Stata Statistical Software: Release 15. College Station, TX: StataCorp LLC).

## Results

### Patients and tumor characteristics

**Fig. 1:**
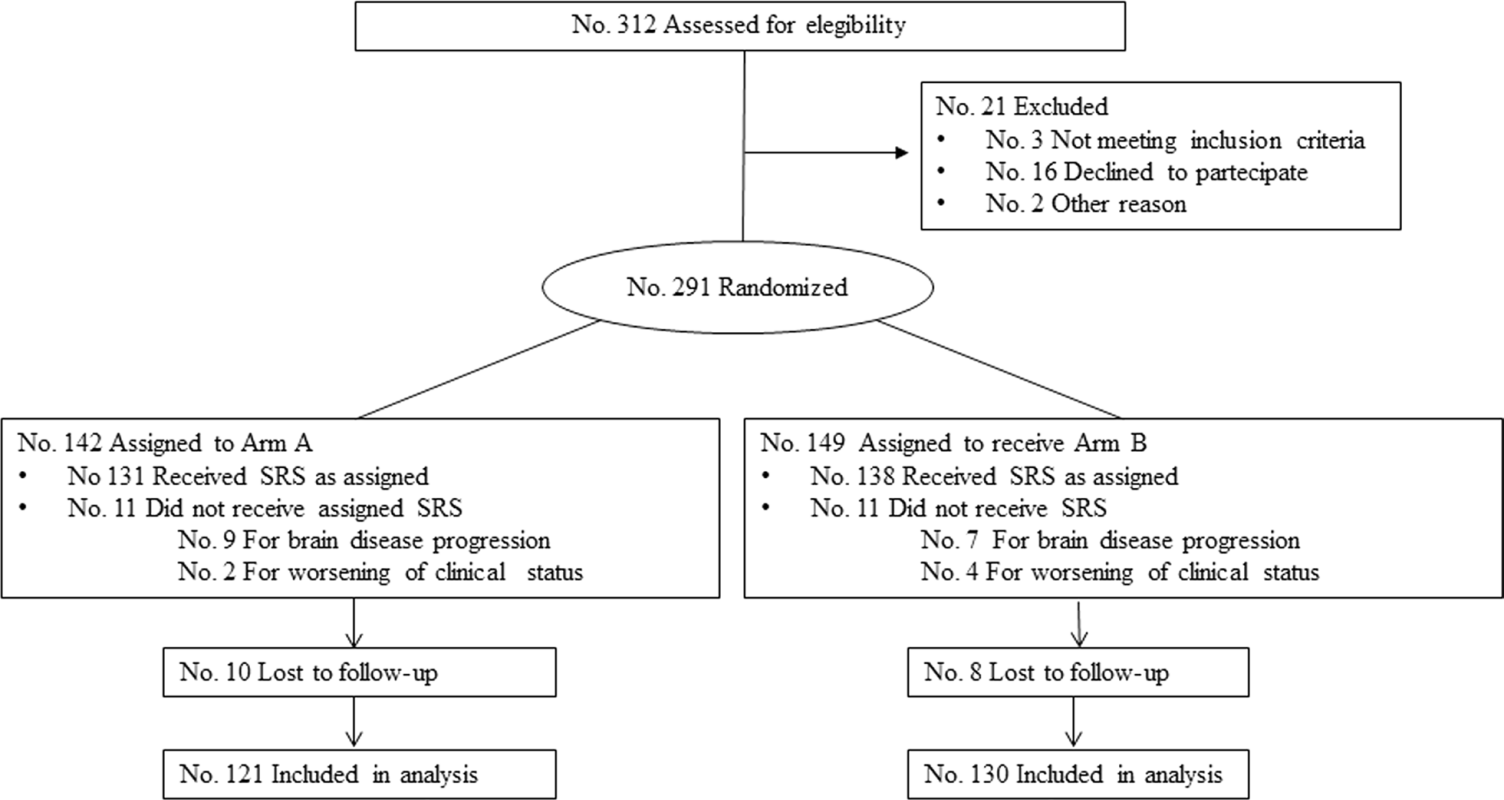
CONSORT flow diagram of patients enrolled in the randomized trial comparing Arm-A and Arm-B

Between October 2014 and June 2020, 251 patients for 449 treated BMs were enrolled and randomly assigned to Arm-A (121) or Arm-B (130), as shown in Fig. 1.

The total number of eligible patients was 312, and 19.5% were excluded for the reasons outlined in Fig. 1. The median follow-up time was 16.9 months (range 1.8–80.9 months) for all patients and 42.0 months (range 11.4–80.9 months) for the alive patients. Baseline patient and tumor characteristics, stratified for SRS modality, are shown in Table 1.

**Table 1:** Baseline patient, and tumor characteristics stratified for radiosurgery (SRS) modality

	Whole cohort No. pts (%)	Arm-A No. pts (%)	Arm-B No. pts (%)
	251 (100)	121 (48.2)	130 (51.8)
Median age [years (range)]	63 (37–84)	62 (37–83)	63 (37–84)
Gender			
Male	139 (55.0)	68 (56.2)	71 (54.6)
Female	112 (45.0)	53 (43.8)	59 (45.4)
KPS			
70	13 (5.2)	6 (5.0)	7 (5.4)
80	35 (13.9)	19 (15.7)	16 (12.3)
90–100	203 (80.9)	96 (79.3)	107 (82.3)
Tumor histology			
NSCLC	143 (57.0)	66 (54.6)	77 (59.2)
Breast cancer	39 (15.5)	23 (19.0)	16 (12.3)
Malignant melanoma	24 (9.6)	8 (6.6)	16 (12.3)
RCC	25 (10.0)	13 (10.7)	12 (9.2)
Other (GI/HN/HCC/GU)	20 (8.0)	11 (9.1)	9 (6.9)
Stage at diagnosis			
I–III	118 (47.0)	59 (48.8)	59 (45.4)
IV	133 (53.0)	62 (51.2)	71 (54.6)
Extracranial metastases	39 (15.5)	17 (14.1)	22 (16.9)
Brain metastases	42 (16.7)	22 (18.2)	20 (15.4)
Extracranial and brain metastases	52 (20.7)	23 (19.0)	29 (22.3)
EC met at time of SRS			
No	88 (35.1)	46 (38.0)	42 (32.3)
Yes	163 (64.9)	75 (62.0)	88 (67.7)
RPA class			
I	39 (15.5)	23 (19.0)	16 (12.3)
II	212 (84.5)	98 (81.0)	114 (87.7)
Systemic therapy after SRS			
No	62 (24.7)	35 (28.9)	27 (20.8)
Yes	189 (75.3)	86 (71.1)	103 (79.2)
Chemotherapy	89 (35.5)	38 (31.4)	51 (39.2)
Targeted therapy	51 (20.3)	28 (23.1)	23 (17.7)
Immune therapy	38 (15.1)	14 (11.6)	24 (18.5)
Hormonal therapy	11 (4.4)	6 (5.0)	5 (3.8)

No., Number; pts, Patients; SRS, Radiosurgery; KPS, Karnofsky performance scale; NSCLC, Non small cell lung cancer; RCC, Renal carcinoma cancer; GI, Gastrointestinal; HN, Head and neck; HCC, Hepatocellular carcinoma; GU, Genitourinary; EC met, Extracranial metastases; RPA, Recursive partial analysis

The number of BMs treated was 236 (53.6%) in Arm-A and 213 (47.4%) in Arm-B. Baseline BMs characteristics stratified for SRS modality are shown in Table 2.

**Table 2:** Brain metastases (BMs) characteristics stratified for radiosurgery treatment arm

	Whole cohort Nbr. Pats. (%)	Arm-A Nbr. Pats. (%)	Arm-B Nbr. Pats. (%)
Total Nbr of metastases	449 (100)	236 (52.6)	213 (47.4)
Nbr of metastases per patient			
1	134 (53.8)	56 (47.1)	78 (60)
2	60 (23.5)	30 (24.0)	30 (23.1)
3	32 (12.8)	19 (15.7)	13 (10)
4	25 (9.9)	16 (13.2)	9 (6.9)
BM localization			
Supratentorial	339 (75.5)	167 (70.8)	172 (80.8)
Intratentorial	100 (22.3)	59 (25.0)	41 (19.2)
Brainstem	10 (2.2)	10 (4.2)	0 (0.0)
Max. diameter			
< 1 cm	217 (48.3)	129 (54.7)	88 (41.3)
1–2 cm	193 (42.8)	87 (36.4)	106 (49.8)
> 2–3 cm	39 (8.9)	20 (8.9)	19 (8.9)

BMs, Brain metastases; No., Number; SRS, Radiosurgery

The median GTV was 0.38 cm^3^ (range 0.002–13.61 cm^3^) and 0.79 cm^3^ (range 0.2–13.8 cm^3^) in Arm-A and Arm-B, respectively, while in Arm-B the PTV was 2.73 cm^3^ (range 0.2–26.7 cm^3^).

The dose prescription was 20 Gy in 51 (21.6%) and 24 Gy in 185 (78.4%) lesions in Arm-A, while it was 24 Gy for all 213 treated lesions in Arm-B. The summary of the dosimetric parameters is given in Additional file 1: Table S1. Planning constraints on OARs were met for all organs. Arm-A data showed a significantly lower healthy brain irradiation. Concerning targets, the near-to-maximum dose in the Arm-A resulted 1.7 times higher than in Arm-B. The conformity and homogeneity indexes resulted better for Arm-B, while the dose gradient was sharper for Arm-A.

### Outcomes

Table 3 summarizes the RN (G2-G3) distribution for the whole cohort and the two trial arms including details about the target volume (cm^3^) and delivered dose.

**Table 3:** BMs characteristics in relation to the occurrence of symptomatic radionecrosis stratified for SRS modality

	Whole cohort	Arm-A	Arm-B
Symptomatic RN (G2-G3)	56	29	27
Median occurrence time months (range months)	10.3 (1.2–54.8)	6.9 (1.2–32.3)	15.9 (4.9–54.8)
Median volume cm^3^ (range cm^3^)	3.1 (0.04–13.6)	2.7 (0.04–13.6)	3.2 (1.1–13.6)
G2 RN	44	24	20
Median occurrence time months (range months)	9.9 (1.2–54.8)	7.5 (1.2–32.3)	14.2 (4.9–54.8)
Median volume cm^3^* (range cm^3^)	3.2 (0.04–13.6)	2.5 (0.04–10.0)	4.8 (1.1–13.6)
Doses delivered			
20 Gy	10	10	0
24 Gy	34	14	20
G3 RN	12	5	7
Median occurrence time months (range months)	24.1 (2.5–44.9)	3.9 (2.5–14.6)	35.6 (16.5–44.9)
Median volume cm^3^* (range cm^3^)	2.0 (0.6–13.6)	3.4 (0.6–13.6)	1.9 (1.1–13.6)
Doses delivered			
20 Gy	1	1	0
24 Gy	11	4	7
Predictors of symptomatic RN occurrence			
Maximum BM diameter	*p* << 0.001 HR 2.2 [95% CI 1.6–3.1]	*p* << 0.001 HR 4.4 [95% CI 2.5–7.8]	*p* = 0.059 HR 1.8 [95% CI 1.0–3.4]
Target volume	*p* << 0.001 HR 1.1 [95% CI 1.0–1.2]	*p* << 0.001 HR 1.4 [95% CI 1.3–1.5]	*p* = 0.076 HR 1.1 [95% CI 1.0–1.1]

SRS, Radiosurgery; RN, Radionecrosis; G2, Grade two; G3, Grade three; SRS, Radiosurgery

*At BMs treatment time

The 1-year RN incidence was 6.7% (95% CI 4.7–9.4%) in the whole cohort, 9.3% (95% CI 6.2–13.8%) in Arm-A and 3.8% (95% CI 1.9–7.4%) in Arm-B without statistically significant differences between the 2 arms (*p* = 0.43). A graphical representation of CIRM is provided in Additional file 2: Fig. S2A. All 12 patients with grade 3 RN underwent planned surgical resection. In all cases a 100% RN has been recorded. The treatment effect was judged only in cases of 0% residual disease. No grade 4 RN has been recorded. Predictive factors for the occurrence of symptomatic RN in the whole cohort and Arm-A were the maximum diameter of the BMs and the irradiated target volume. These did not result significant for Arm-B resulting in a *p* value above 0.05. Concerning absolute occurrence for the whole cohort, local recurrence occurred in 14 (3.1%) cases, and BDF occurred in 104 (41.4%) patients. At the last observation time, 69 (27.5%) patients were alive and 182 (72.5%) dead. Table 4 summarizes the median time and the 1,2,3-year rates for CILP, DBF, DFS, and OS. A graphical representation is reported in Additional file 2: Fig. S2B and Additional file 3: S3. Statistically significant factors influencing the various outcome metrics for the whole cohort and the two arms are also reported.

**Table 4:** Cumulative incidence local progression (CILP), distant brain failure (DBF), disease free survival (DFS) and overall survival (OS)

	Whole cohort	Arm-A	Arm-B	*p*
Cumulative incidence local progression				
Median time (months)	n.r	n.r	n.r	–
1-year (%)	1.8 (0.9–3.5)	0.4 (0.1–3.0)	3.3 (1.6–6.8)	–
2-year (%)	2.5 (1.4–4.4)	0.9 (0.2–3.4)	4.2 (2.2–8.0)	–
3-year (%)	2.7 (1.6–4.8)	1.4 (0.4–4.2)	4.2 (2.2–8.0)	0.0581
Distant brain failure				
Median time (months)	24.9 (18.4-n.r.)	24.9 (15.6-n.r.)	24.4 (14.1-n.r.)	–
1-year (%)	36.5 (30.3–43.4)	35.5 (26.9–45.8)	37.5 (29.1–47.3)	–
2-year (%)	48.4 (41.4–56.0)	48.2 (38.2–59.4)	48.9 (39.4–59.4)	–
3-year (%)	56.2 (48.4–64.2)	52.5 (41.7–64.2)	59.8 (48.9–71.0)	0.3595
Disease free survival				
Median time (months)	7.3 (6.3–8.8)	7.8 (5.9–10.5)	6.9 (6.0–8.4)	–
1-year (%)	31.0 (25.4–36.8)	32.1 (24.0–40.5)	29.2 (21.7–37.1)	–
2-year (%)	17.6 (13.1–22.5)	18.3 (12.0–25.8)	16.9 (11.0–23.8)	–
3-year (%)	14.3 (10.1–19.0)	14.0 (8.3–21.2)	14.4 (9.0–21.1)	0.5960
Overall survival				
Median time (months)	17.8 (15.6–22.8)	18.6 (15.0–25.9)	16.2 (13.3–23.0)	–
1-year (%)	65.7 (59.5–71.2)	65.2 (56.0–73.0)	65.3 (56.3–72.8)	–
2-year (%)	41.1 (34.9–47.1)	42.3 (33.3–51.1)	39.1 (30.7–47.4)	–
3-year (%)	30.0 (24.2–36.0)	29.9 (21.3–38.9)	29.0 (21.4–37.1)	0.4087
Predictors influencing cumulative incidence local progression				
Target volume	*p* < 0.001 HR 1.1 [1.1–1.2]	*p* = 0.499 HR 1.1 [0.8–1.6]	*p* = 0.004 HR 1.1 [1.0–1.2]	–
Predictors influencing distant brain failure				
Number of lesions	*p* = 0.01 HR 1.3 [1.1–1.5]	*p* < 0.01 HR 1.5 [1.1–1.9]	0.42 HR 1.1 [0.8–1.5]	–
Predictors influencing disease free survival				
Patients age	*p* = 0.001 HR 1.02 [1.01–1.03]	*p* = 0.029 HR 1.02 [1.00–1.04]	*p* = 0.022 HR 1.02 [1.00–1.04]	–
KPS	*p* = 0.001 HR 0.7 [0.5–0.8]	*p* = 0.003 HR 0.6 [0.4–0.8]	*p* = 0.058 HR 0.71 [0.5–1.0]	–
RPA class	*p* = 0.003 HR 1.8 [1.2–2.7]	*p* = 0.007 HR 2.1 [1.2–3.6]	*p* = 0.154 HR 1.5 [0.9–2.7]	–
Number of lesions	*p* = 0.017 HR 1.2 [1.0–1.3]	*p* = 0.089 1.2 [1.0–1.4]	*p* = 0.052 1.2 [1.0–1.5]	–
Predictors influencing overall survival				
Patients age	*p* < 0.001 HR 1.03 [1.01–1.04]	*p* = 0.030 HR 1.02 [1.00–1.05]	*p* = 0.002 HR 1.03 [1.01–1.05]	–
KPS	*p* = < 0.001 HR 0.5 [0.4–0.7]	*p* < 0.002 HR 0.6 [0.4–0.8]	*p* < 0.001 HR 0.5 [0.3–0.7]	–
RPA class	*p* = 0.05 HR 1.8 [1.2–2.7]	*p* = 0.106 HR 1.6 [0.9–2.9]	*p* = 0.046 HR 2.0 [1.0–3.8]	–
Number of lesions	*p* = 0.002 HR 1.3 [1.1–1.4]	*p* = 0.018 HR 1.3 [1.0–1.5]	*p* = 0.031 HR 1.3 [1.0–1.6]	–

Values in brackets are the 95% CI

Among patients with local progression, 7 out of 14 underwent HSRS, and 1 WBRT because of concomitant BDF. Considering the 104 patients with BDF, 89 patients received further RT treatment: 68 received SRS or hypofractionated stereotactic radiosurgery (HSRS) for limited (1–4 BMs) brain progression, and 21 received WBRT for meningeal or diffuse parenchymal progression. No clinically significant differences were seen over time until cranial or extra-cranial disease progression, comparable in both arms. KPS did not change from baseline during the observation time. No severe toxicities have been recorded.

## Discussion

The results of a randomized phase 3 trial comparing two SRS treatment approaches using different platforms, each characterized by specific features, were reported. The main differing characteristics among the two arms are: (i) the use of a rigid frame system in one and a frameless in the other; (ii) the employ of margins from the GTV in one arm only; (iii) the different dose prescriptions. Concerning the use of the frame, although more modern versions of the multi-source gamma rays system allow the frameless treatments, this is not the usual practice applied, while, on the linacs, the use of masks instead of frames is the most employed mode of patient immobilization in clinical practice. The PTV generation, applying margins from GTV, is, once more related to historical procedures, related to the older linacs technologies, in which the mechanical precision was lower than what is currently available. Nevertheless, the study, which started seven years ago, applied the clinical practice at the design time and was not modified. The evolution of the linac-based techniques would allow today the application of smaller or even zero margins. Such practice is ongoing also in our institute for patients treated out of the protocol but was not included for obvious reasons. However, no increasing RN has been observed despite larger volumes being handled in Arm B. Finally, a two-level dose prescription was also foreseen in the linac arm as per the protocol. In practice, the lesion diameter of the larger metastases in the Arm-B was modestly exceeding the threshold except for one lesion in a multiple lesion patient. This was far from any organ at risk. Since in all cases no dose-volume parameters were at risk for those patients if treated at full dose. For this reason, the Arm-B does not include any patient treated with 20 Gy; we acknowledge this as a minor protocol violation. We also acknowledge that most clinical centers exclusively use one device or the other according to availability. Nevertheless, the availability of the two in a single institute allowed this trial although a better data analysis consistency would be in a multi-institutional investigation. About 20% of eligible patients were excluded from the analysis for the patient cohort for various reasons. This strategy is a formal deviation from the trial protocol, which assumed an intention-to-treat analysis. Nevertheless, due to this significant rate and to minimize potential negative biases, we opted for their exclusion, which is also supported by the fact that most patients did not receive any radiosurgery treatment. We acknowledge this as a protocol deviation and a limitation of the study. The study’s primary endpoint was the RN assessment, assuming a superiority of linac-based SRS compared to the gamma-ray platform. Published data, although not randomized but only comparative, showed a comparable local control using the two modalities for SRS, but a higher risk of RN employing gamma-ray platform compared to linac-based SRS, above all when large BMs are treated, has been recorded [23, 25, 31–33]. Considering the availability in our institution of the two modalities for SRS, we wanted to verify, in a randomized matter, the benefits and potential risks of them. The trial failed to reach the primary objective, as it did not show the linac arm's superiority in reduction of RN incidence and helped to confirm the similarities between the two approaches. The inherent planning differences in the arms, particularly for volumes and dosing, unfavorable in Arm B for adding margins to generate PTV, and the higher doses employed (24 Gy in all cases), might have influenced the comparison or minimized the observed difference in the necrosis incidence. Despite this, we found a higher occurrence, although not statistically significant, of symptomatic RN cumulative incidence by one year in Arm-A with respect to Arm-B, 3.8% *vs* 9.3%, which occurred earlier in Arm-A compared to Arm-B, 6.9 months, and 15.9 months, respectively. As expected, the maximum tumor diameter of BMs and the target volume irradiated has proven to be a significant factor influencing the occurrence of symptomatic RN (grade 2–3). Notwithstanding a larger volume has been irradiated in Arm-B, for the addition of isotropic margins to generate planning target volume (PTV) from the gross tumor (GTV) ones, unlike Arm-A where no margins were employed, the target volume significantly affected the onset of RN in Arm-A only with a *p* value << 0.001 (Table 3). Besides, the lesion volume was not part of the randomization process, and some imbalance in the size distribution between the two arms occurred and might have further masked the difference in the expected rate of RN. The appearance of grade 3 RN, requiring an invasive therapeutic approach, such as surgical resection, was anyhow earlier in Arm-A (median time 3.9 months) compared to Arm-B (median time 35.6 months). This data is probably related to the delivery modality in Linac arm (at the mean dose to PTV), and the Volumetric Modulated Arc Therapy technique used, compared to the inhomogeneity of the dose prescribed in GK arm (at isodose line 50%) with the higher central dose. To our knowledge, this is the first randomized trial comparing two modalities for radiosurgery in terms of safety and efficacy. Published data comparing the two modalities for SRS came from subset analyses of prospective trials. The RTOG 9508, which evaluated WBRT with or without SRS boost, showed similar results among patients treated with GK and LINAC [15]. Similarly, the RTOG 9005, aiming to find the maximum tolerated dose of single-fraction SRS in patients with previously treated brain tumors, did not observe any association of treatment unit with central nervous system (CNS) toxicity [23, 24]. A recent multi-institutional study assessed outcomes in patients treated with linacs and multi-source gamma-ray units. After propensity score matching, a similar overall survival has been recorded with a lower incidence of radionecrosis in patients treated with linacs [31]. The development of brain RN is the most common complication of SRS, ranging from 6% to more than 30% of treated lesions, occurring, in most studies, from < 6 months to several years after SRS, with a median time to onset of roughly a year [25, 32–35]. Factors related to the development of radionecrosis after SRS include histology of the primary tumor, the association with systemic therapy, BMs location, total dose delivered, treated volume, and volume of the brain receiving a specific dose [25, 31, 32]. In our series, no other factors were recorded as conditioning RN except for the volume of BMs treated. The emerging data was that symptomatic RN occurred more frequently in BM larger than 1 cm in maximum diameter, particularly if receiving higher SRS doses. Instead, all cases of G3 RN occurred for BMs of about 2 cm receiving 24 Gy, suggesting that a dose reduction is needed in these cases. Comparable brain control has been obtained, achieving 95% of BMs treated, confirming the same effectiveness of the two radiosurgery modalities. The median OS time was 18 months, with more than 60% of patients alive at one year, without statistically significant differences between the two arms. From a dosimetric point of view, the multi-source gamma-ray unit showed rapid fall-off dose close to the tumor; the linac-based plans were associated with a better conformality (for both indexes) and homogeneity. Treatment efficiency favored the linac-based treatments in terms of beam-on time. The total treatment time was not quantified for all cases, but using a frame for the Arm-A cases resulted in longer preparation times than the Arm-B. The trial has several limitations related to the different technologies employed, the various SRS doses delivered (discussed above), and the different stratification regarding the number of BMs treated in the two arms. This latter was related to the evidence of a higher number of lesions observed at MRI performed on the day of treatment while respecting the trial inclusion criteria. Among other limits, we recognize that the dose constraint applied for the brain stem was more relaxed if compared, e.g. to the AAPM TG-101 recommended value of ${\text{D}}_{{0.5{\text{cm}}^{3} }}$ ≤ 10 Gy for single-dose treatments. The chosen constraint was derived from our clinical practice at the time of trial design. A cross-check on the data from Arm-B showed that ${\text{D}}_{{0.5{\text{cm}}^{3} }}$ = 4.3 ± 4.4 Gy, consistent with the more stringent constraints. As a final note, a preliminary interim analysis was performed in 2018. Although not explicitly foreseen by the trial protocol, it did not interfere with any procedures and did not alter the treatment arms in any manner. Using the Lan–DeMets method, we calculated the modified alpha level induced by the introduction of the interim analysis and leading to a figure of 0.038 instead of the initial 0.05. Indeed, this value did not show any significant impact on our conclusions since there were not *p* values above 0.038 and less than 0.05 except RPA class in OS, that was not considered as a significant prognostic factor in this study.

## Conclusion

Given the technical differences between the treatment platforms investigated in this single-institution study, linac-based SRS (Arm-B) did not lead to significantly lower rates of grade 2–3 RN versus the multi-source gamma-ray system (Arm-A) in a population of patients with limited brain metastases of limited volume. No significant difference in patients outcome was observed between both arms. Multicentric prospective randomized trials are needed to confirm this data further.

## Supplementary Information

**Additional file 1. Table S1.** Summary of dosimetric data for the targets and the organs at risk for the two arms.

**Additional file 2. Fig. S2.** Cumulative incidence of radionecrosis (**A**) and and local progression (**B**) for GK (Gamma Knife, solid line) and Linac-based radiosurgery (dashed line).

**Additional file 3. Fig. S3.** Kaplan–Meyer plots for progression free survival (**A**), overall survival (**B**) and Brain Distant failure (**C**) for GK (Gamma Knife, solid line) and Linac-based radiosurgery (dashed line). Vertical bars, censored cases.

## Data Availability

Research data are store in an institutional repository and will be shared upon request to the corrisponding author.

## Abbreviations

SRS: Radiosurgery
BMs: Brain metastases
CIRN: Cumulative incidence of radionecrosis
CILP: Cumulative incidence of local progression
BDF: Brain distant failure
DFS: Disease free survival
OS: Overall survival
CI: Confidence interval
HR: Hazard ratio
WBRT: Whole brain radiation therapy
Gy: Gray
KPS: Karnofsky performance status
RPA: Recursive partitioning analysis
DS-GPA: Disease specific-grade prognostic assessment
SCLC: Small cell lung cancer
CTCAE: Common Terminology Criteria for Adverse Events
MRI: Magnetic resonance image
11CMETPET: Carbon metionine positron emission thomography
RANO: Response Assessment in Neuro-Oncology
GTV: Gross tumor volume
PTV: Planning target volume
IGRT: Image guided radiation therapy
CBCT: Cone beam computer thomography
RTOG: Radiation therapy oncology group
CI: Conformity index
