# Supplementary material for: Radiosurgery of limited brain metastases from primary solid tumor: results of the randomized phase III trial (NCT02355613) comparing treatments executed with a specialized or a C-arm linac-based platform
Source: Radiat Oncol. 2023 Feb 7;18:28. doi: 10.1186/s13014-023-02216-5 (PMC9906937; doi:10.1186/s13014-023-02216-5)
Supplement: Supplementary file 1 — Additional file 1. Table S1. Summary of dosimetric data for the targets and the organs at risk for the two arms. [file 13014_2023_2216_MOESM1_ESM.docx]

Table S1. Summary of dosimetric data for the targets and the organs at risk for the two arms.

| **Metric** | **Aim** | **Arm-A** | **Arm-B** | **p** |
| --- | --- | --- | --- | --- |
| **Targets** | | | | |
| **CI RTOG** | 1 | 3.35±4.58 | 1.17±0.46 | <0.001 |
| **PCI** | 1 | 0.55±0.28 | 0.86±0.41 | <0.001 |
| **GI** | Minimize | 3.03±0.73 | 4.24±1.25 | <0.001 |
| **HI** | Minimize | 0.66±0.13 | 0.20±0.21 | <0.001 |
| **D_1%_** |  | 42.05±5.58 | 25.03±1.04 |  |
| **Target coverage** | Maximize [%] | 99.7±0.8 | 98.1±2.1 | <0.001 |
| **Prescription isodose** | - | 50% isodose | Mean dose to target | - |
| **Organs at risk** | | | | |
| **Healthy brain** | V_12Gy_ : minimize [cm^3^] | 2.9±3.3 | 18.3±19.1 | <0.001 |
|  | V_5Gy_: minimize [cm^3^] | 16.3±18.6 | 72.4±46.0 | <0.001 |
| **Brain Stem** | V_12Gy_ ≤ 10 cm^3^ [cm^3^] | 0.14±0.84 | 0.03±0.15 | 0.08 |
| **Optical nerves** | D_2%_ ≤8 Gy [Gy] | 0.93±2.11 | 0.59±1.2 | 0.10 |
| **Chiasm** | D_2%_ ≤12 Gy [Gy] | 0.87±1.11 | 1.07±1.6 | 0.30 |
| **Cochlea left** | Mean dose ≤12 Gy [Gy] | 0.42±0.57 | 0.72±1.16 | 0.05 |
| **Cochlea right** | Mean dose ≤12 Gy [Gy] | 0.39±0.57 | 0.56±0.78 | 0.01 |

CI RTOG: RTOG Conformity Index; PCI: Paddick Conformity Index; GI: Gradient Index; HI: Homogeneity Index; V_xGy_: volume receiving at least X Gy; D_X%_: dose received by at most X% of the volume
