# Supplementary figures and images for: Radiosurgery of limited brain metastases from primary solid tumor: results of the randomized phase III trial (NCT02355613) comparing treatments executed with a specialized or a C-arm linac-based platform
Source: Radiat Oncol. 2023 Feb 7;18:28. doi: 10.1186/s13014-023-02216-5 (PMC9906937; doi:10.1186/s13014-023-02216-5)

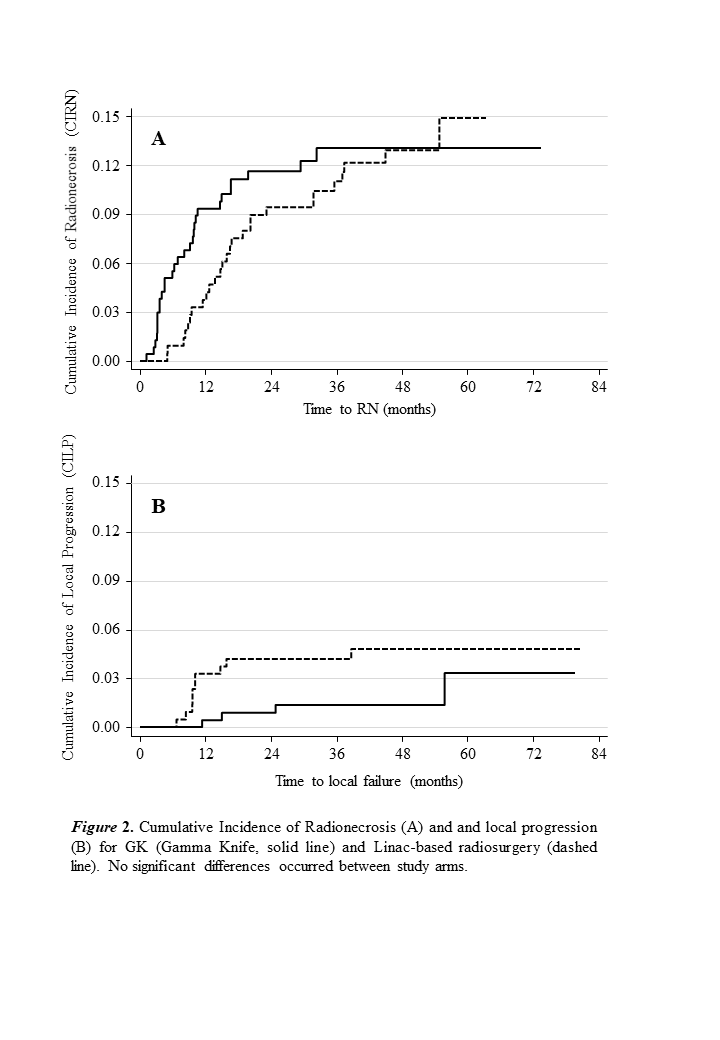

Supplement: Supplementary file 2 — Additional file 2. Fig. S2. Cumulative incidence of radionecrosis (A) and and local progression (B) for GK (Gamma Knife, solid line) and Linac-based radiosurgery (dashed line). [file 13014_2023_2216_MOESM2_ESM.tif]

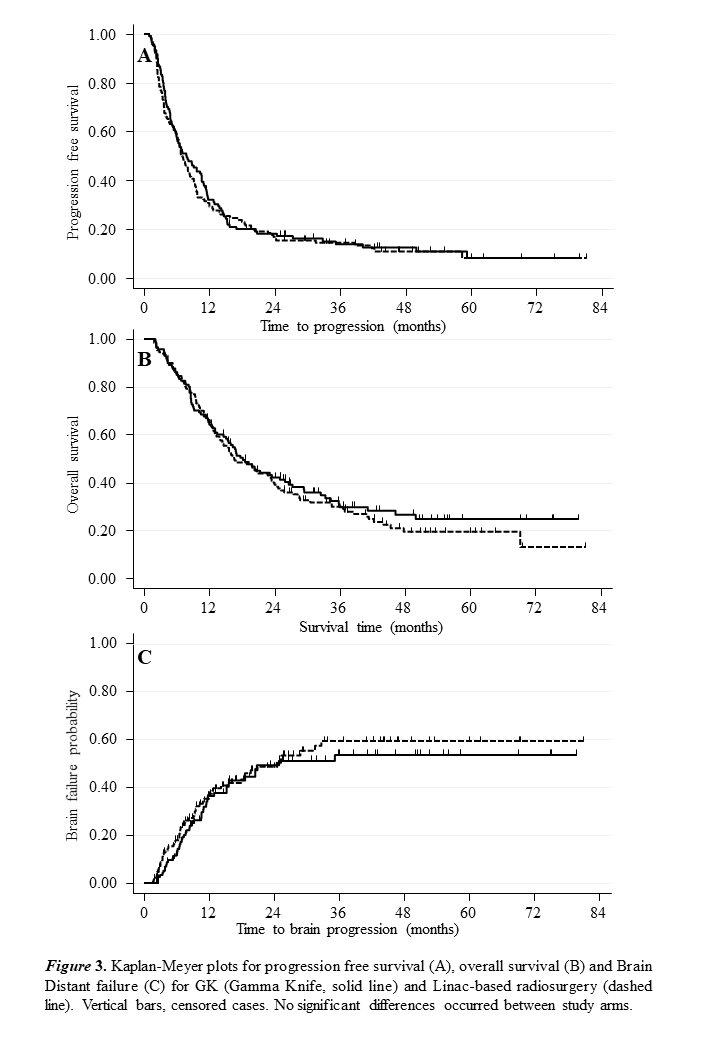

Supplement: Supplementary file 3 — Additional file 3. Fig. S3. Kaplan–Meyer plots for progression free survival (A), overall survival (B) and Brain Distant failure (C) for GK (Gamma Knife, solid line) and Linac-based radiosurgery (dashed line). Vertical bars, censored cases. [file 13014_2023_2216_MOESM3_ESM.tif]
